# ‘Very brief advice’ (VBA) on smoking in family practice: a qualitative evaluation of the tobacco user’s perspective

**DOI:** 10.1186/s12875-020-01195-w

**Published:** 2020-06-24

**Authors:** Sophia Papadakis, Marilena Anastasaki, Maria Papadakaki, Μaria Antonopoulou, Constantine Chliveros, Chrissi Daskalaki, Dionisis Varthalis, Sofia Triantafyllou, Irene Vasilaki, Andy McEwen, Christos Lionis

**Affiliations:** 1 Clinic of Social and Family Medicine, Faculty of Medicine, University of Crete, P.O. Box: 2208, 71003 Heraklion, Crete Greece; 2 Division of Prevention and Rehabilitation, University of Ottawa Heart Institute, Ottawa, Ontario Canada; 3 Department of Social Work, School of Health Sciences, Hellenic Mediterranean University, Heraklion, Crete Greece; 4 Spili Primary Care Centre, Rethymno, Crete Greece; 5 Agia Fotini Rural Practice, Rethymno, Crete Greece; 6 Perama Primary Care Centre, Rethymno, Crete Greece; 7 1st Local Healthcare Unit, Heraklion, Crete Greece; 8 Viannos Primary Care Centre, Heraklion, Crete Greece; 9 National Centre for Smoking Cessation and Training, Dorchester, UK

**Keywords:** Smoking cessation, Primary care, Very brief advice, Patient perspective, Qualitative research

## Abstract

**Background:**

Very Brief Advice on smoking (VBA) is an evidence-based intervention designed to increase quit attempts among patients who smoke. VBA has been widely disseminated in general practice settings in the United Kingdom, however its transferability to Southern European settings is not well established. This study sought to document the perspectives of Greek general practice patients in terms of the acceptability and satisfaction with receiving VBA from their general practitioner (GP) and its influence on patients’ motivation to make a quit attempt. We also examine patient identified barriers and facilitators to acting on VBA.

**Methods:**

Semi-structured interviews were conducted with 50 patients who reported current tobacco use recruited from five general practices in Crete, Greece. All patients received VBA from their GP and interviews were conducted immediately after the GP appointment. Thematic analysis was used to analyze data.

**Results:**

The majority of patients were satisfied with the VBA intervention. Approximately one quarter of patients reported they were motivated to make an attempt to quit smoking after receiving VBA from their GP. Patients identified a clear preference for VBA to be delivered in a supportive manner, which communicated genuine concern versus fear-based approaches. Patients with an existing smoking-related illness were more likely to report plans to act on their GP’s VBA. Patients not ready to quit smoking indicated they would be likely to seek the support of their GP for future quit attempts as a result of VBA. Many patients reported low self-efficacy with quitting and apprehension about available quit smoking supports.

**Conclusions:**

VBA was positively received by the majority of smokers interviewed. Participating patients confirmed the motivational role of advice when delivered in a supportive and caring manner. Personal health status, beliefs about quit smoking supports, and low self-efficacy appear to influence patient’s motivation to make an aided quit attempt.

## Background

Smoking is the leading preventable cause of death and disability in Europe and reducing tobacco use is a priority of the World Health Organization European region, international treaties, as well as the national governments in many European countries [1, 2]. In many European countries rates of quitting among smokers is low or modest [3, 4]. Health care professionals have an important role to play in triggering quit attempts among patients who smoke and supporting cessation among those patients ready to quit [5–8]. Research has found patients who receive advice and an offer of support with quitting from their general practitioner (GP) are more likely to make an attempt to quit smoking and be successful with quitting in the long-term [5, 6]. There is strong evidence that a combination of behavioural counseling and pharmacotherapy can increase success with quitting, however these evidence-based treatments are often not used as part of quit attempts [3, 9]. There is an existing body of literature, which has examined barriers to quitting, as well as, use of available stop smoking treatments among smokers. Nicotine addiction, stress, a smoker’s belief that they will not be able to quit (self-efficacy), and the expectation that stop-smoking support is ineffective or will not meet their needs, time constraints or issues with access, have all been cited as barriers [10–17].

**Fig. 1:**
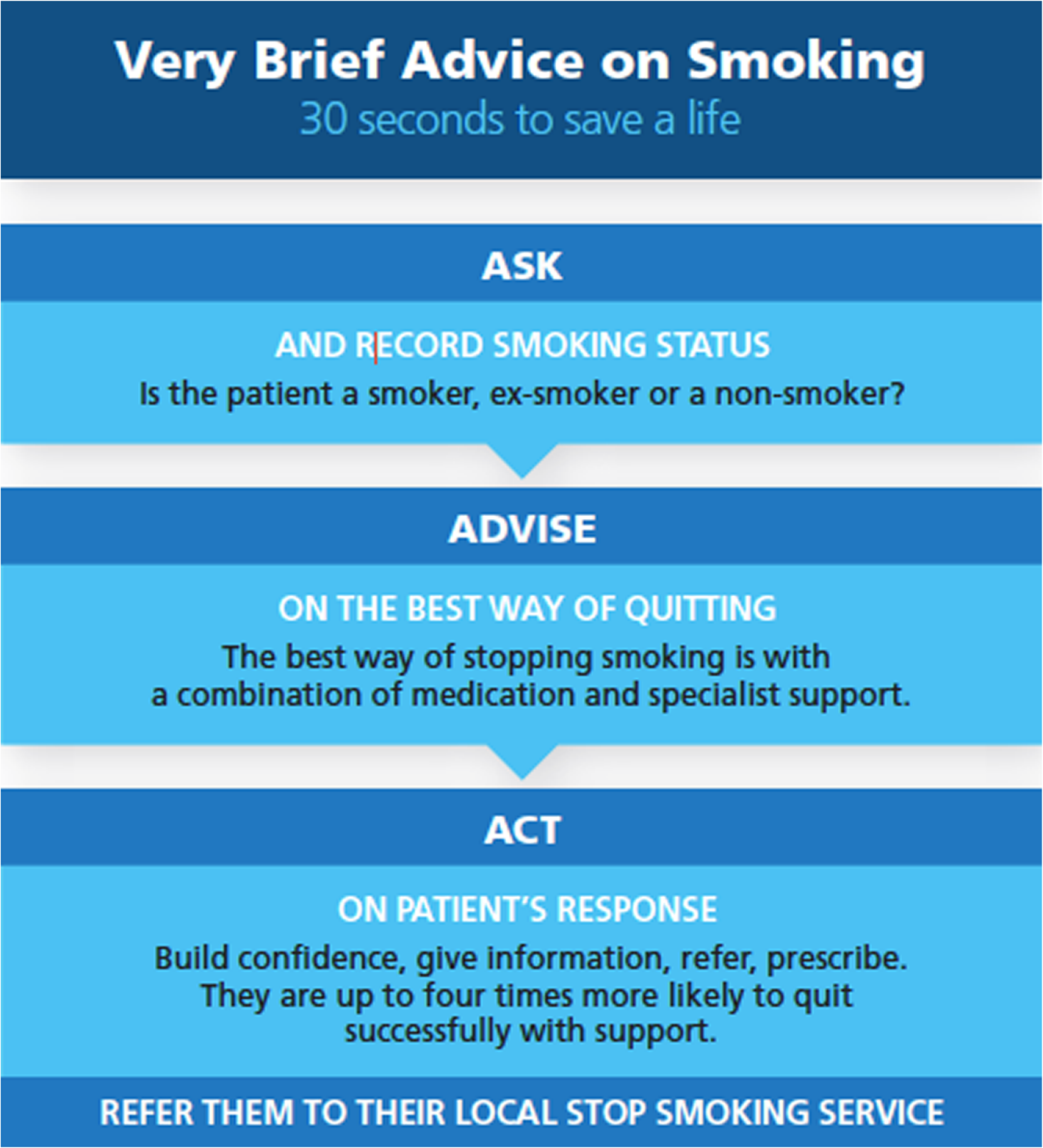
Very Brief Advice on Smoking intervention

Very Brief Advice on smoking (VBA) is an evidence-based intervention designed to increase quit attempts among people who smoke [6, 18]. VBA was developed by the National Centre for Smoking Cessation Training (NCSCT) in the United Kingdom (UK) (www.ncsct.co.uk/VBA) and involves three steps: “Ask” patients about their tobacco use, “Advise” them that the best method of quitting is with a combination of medication and behavioural support, and “Act” by supporting them with making a quit attempt using available cessation supports (Fig. 1). VBA has been designed based on the COM-B theoretical model for behavior change and is designed to deliver effective advice without taking up too much time or harming relationships with patients [18, 19]. If a smoker is interested in quitting, they are offered support and medication wherever it is locally available. VBA does not attempt to deliver more intensive counselling techniques to persuade smokers who are not ready to quit, but rather assumes that the VBA intervention, if repeated, will increase the patient’s likelihood to quit. VBA has primarily been delivered in the UK where more than 60,000 health care professionals have completed the training.

Greece has among the highest rates of smoking in Europe (36% vs. 24%), as well as the highest rates of daily cigarette consumption [4]. In contrast to the UK, a very small proportion (12%) of Greek smokers made an attempt to quit smoking in the past year and almost 44% report they have never attempted to quit smoking in comparison to almost 70% in the UK [4]. Greece has among the poorest ranking among EU countries in terms of investment made in tobacco control policy and cessation, lagging behind many European countries in implementing policy-based interventions such as smoke-free public spaces and public education campaigns designed to de-normalize smoking [3, 4, 20]. In contrast to the well-developed cessation system in the UK, there are no active national public education campaigns promoting cessation and specialist quit smoking clinics are primarily found only in large city centres that, by in large, operate on a fee-for-service basis [3]. In Greek general practice settings, modest rates of cessation advice (51%) have been reported, while rates of assistance with quitting are also very low (16%) [4, 21].

It is not known if VBA will be acceptable and effective in increasing quit rates among smokers identified in general practice setting in Greece. In particular, there is a limited understanding in terms of cultural, smoking-related, or other factors that may affect the acceptability and effectiveness of VBA among patients identified in Greek general practice settings compared to the UK. The aim of this study was to document the perspectives of Greek general practice patients in terms of the acceptability and satisfaction with receiving VBA from their GP and its influence on patients’ motivation to make a quit attempt. We also examine the patient-identified barriers and facilitators to acting on VBA. Findings of this study may inform future policy and practice in terms of the role of VBA in Greece and other similar European settings.

## Methods

### Study design

During the first half of 2018, we conducted a qualitative study involving semi-structured interviews with 50 patients who smoked recruited from GP practices. The interviews were conducted immediately following the GP appointment and explored patients’ views regarding VBA, their intention to quit smoking, as well as facilitators and barriers in terms of acting on VBA. The study received ethics approval from the local research ethics board at the Regional Health Authorities of Crete (Protocol Number: 13683 / 09–08-17). All participating providers and patients signed an information sheet and consent form. The consolidated criteria for reporting qualitative research (COREQ) was used to report on study findings [22].

### Setting and participants

The study was conducted in a sample of five GP practices on the island of Crete in Greece (representing 10% of GPs in the region sampled). From each GP’s practice ten eligible patients were recruited. Patients were eligible to participate in the study if they met the following criteria: were current smokers (≥1 cigarette per day on most days of the week); were 18 years of age or older; were scheduled for an annual exam or non-urgent medical appointment; were able to read and/or understand Greek; and, had the mental capacity to provide informed consent and complete study protocols (i.e. no diagnosis of dementia or other advanced cognitive impairment).

### Theoretical framework

VBA is based on the COM-B (‘capability’, ‘opportunity’, ‘motivation’ and ‘behaviour’) model for behavior change [19]. COM-B is an integrative model, which has been informed by several models of behavior change [19]. The model proposes that for someone to engage in a particular behaviour (B) they must be physically and psychologically capable (C), have the social and physical opportunity (O) to do the behaviour and be motivated (M) to do the behaviour more than any other competing behaviours at that moment [23]. COM-B was used as a guiding framework for the present evaluation.

### Intervention

Prior to the interview all patients received VBA on smoking cessation from their GP. All GPs received a 5-h training session on VBA using locally adapted NCSCT training materials [24]. The VBA intervention followed the 3A approach (Ask-Advise-Act) as follows: a) GP asked patients about their smoking status (Ask); b) advised them to quit smoking and jointly with the patient identified the best way of doing this (Advise); c) acted by offering practical support to patients who communicated an interest in stopping smoking (Act). For the Act component, GPs were trained to offer patients quit smoking pharmacotherapy, refer them to an existing hospital-based smoking cessation service or offer follow-up support arrangements at their practice.

### Procedures

During the data collection period, a research assistant was located in the GP practice waiting room and conducted eligibility screening among consecutive patients presenting for medical consultation. Patients were then invited to take part in an exit interview, carried out in a private location within the practice to assure confidentiality. Eligible patients who agreed to participate in the study, signed the study information sheet and consent form prior to participation. Participants were informed their responses would remain confidential. An exit survey was completed with all consenting patients at the end of their medical consultation. The exit survey gathered demographic information and information on their smoking history. Semi-structured interviews were then conducted with all consenting patients. An interview guide was developed including open-ended questions about patients’ feelings about receiving VBA from their GP (acceptability and satisfaction), their motivation to quit smoking by undertaking any of the recommended smoking cessation treatments, as well as personal barriers and facilitators to acting on the VBA intervention by agreeing to use counseling support (from their GP or by referral to a hospital-based smoking cessation clinic) and/or a quit smoking pharmacotherapy. Two research assistants (MA and IV), who were trained on the study objectives and interview guide by study investigators (SP and MP), conducted the exit interviews. Research assistants were advised to be flexible during the data collection stage in order to identify and extend further on important emerging issues not directly addressed by the guide. The interviews had a mean duration of 15 min. Interviews were audio recorded and field notes were completed. Participants did not have an existing relationship with the research assistants and no other individuals were present during the interview.

### Data analysis

Characteristics (age, gender, cigarettes per day) of participants and non-participants were compared using t-tests and chi-square analysis. Descriptive statistics were used to summarize patient demographics, smoking-related characteristics and recall of VBA delivery. Interview data were transcribed verbatim and translated into English. The raw transcripts generated in the VBA interviews were analysed in order to answer the three research questions. A case description was initially drafted for each of the VBA interview discussions using all data. Then, the process included the coding of data into meaningful groups and establishing a coding scheme. Two persons coded the data (MD, EC) independently and a third one undertook the reconciliation process (MP). Data saturation was assessed amongst the sample based on the consistency of data gathered from participating smokers and was determined to be sufficient. The list of different codes were sorted into potential themes, based on recurring regularities and coherent patterns of meaning [25]. Thematic analysis was guided by the COM-B model and prior research regarding mediators for behavior change among smokers [14, 15, 19, 26]. The initial thematic analysis report was produced for discussion and validation by the research team and is available upon request. This led to re-categorization of some data and development of additional content. We examined the degree to which responses were similar among patient sub-groups (i.e. clustering) including: GP practice, geographic location (urban/rural), motivation to quit smoking, presence of smoking-related illness, as well as, other factors identified in thematic analysis. Project team members also identified the most salient quotes from the thematic analysis. Quotes are identified with the patient ID number.

## Results

During the study recruitment period a total of 134 patients were screened (mean age 55.3 years, 50.7% female, 42.5% currently smoking). Of the 57 patients who reported current tobacco use, 50 patients consented to participate in the study (response rate: 87.7%). Participants and non-participants were similar in age (*p* = 0.424) and gender (*p* = 0.701), however participants were significantly more likely to report greater daily cigarette consumption (non-participants: 14.0 ± 7.2 vs. participants: 24.6 ± 13.3; *p* = 0.040).

### Characteristics of participants

Table 1 presents the characteristics of participants. Participants were primarily male (64.0%) with a mean age of 50.4 years [range: 23–84]. More than a quarter of participants had less than high school education (28.6%) and the majority lived in rural areas (60.0%). One in four participants reported having a smoking-related illness. Participants reported smoking an average of 24.6 (SD ± 13.3) cigarettes per day for a mean duration of 32.4 years (SD ± 13.6). The majority (67.0%) of participants smoked within 30 min of waking in the morning, a proxy for greater nicotine dependence.

**Table 1:** Characteristics of patients

Variable	Value
**Age in years, mean**	50.4 (14.7)
**Age group, n (%)**	
18–24	3 (6.0)
25–39	11 (22.0)
40–54	15 (30.0)
55–64	13 (26.0)
≥ 65–75	8 (16.0)
**Male, n (%)**	32 (64.0)
**Greek nationality, n (%)**	48 (98.0)
**Area of residence, n (%)**	
Urban	10 (20.0)
Semi-urban	10 (20.0)
Rural	30 (60.0)
**Education, n (%)**	
Grade school	14 (28.6)
Junior High school	8 (16.3)
High School	17 (34.7)
College/University	5 (10.2)
Graduate School	5 (10.2)
**Occupation, n (%)**	
Retired	9 (18.0)
Agriculture	9 (18.0)
Business Owner (restaurant, bakery, café)	4 (8.0)
Blue collar employee (cleaner, cook, maintenance, technician, hotel clerk)	10 (10.0)
Dentist, Teacher, Engineer, Accountant	5 (8.0)
Freelancer	2 (4.0)
Public Servant	3 (6.0)
Home maker	3 (6.0)
Unemployed	4 (8.0)
**Medical history**	
Smoking-related illness^a^ n (%)	12 (24.5)
Depression or Anxiety, n (%)	5 (10.2)
Other mental health, n (%)	0 (0)
**Cigarettes per day, mean**	24.6 (13.3)
**Cigarettes per day, n (%)**	
< 15	9 (18.0)
15–20	20 (40.0)
21–40	16 (32.0)
> 40	5 (10.0)
**Time to first cigarette in the morning, n (%)**	
Within 5 min	11 (22.4)
6–30 min	22 (44.9)
31–60 min	5 (10.2)
After 60 min	11 (22.4)
**Age of smoking initiation, mean (41)(41)(40)(40)(40)(40)(39)(38)(38)(37)(36)(35)(34)(33)(33)(33)**	18.2 (6.0)
**Years of smoking, mean (41)(41)(40)(40)(40)(40)(39)(38)(38)(37)(36)(35)(34)(33)(33)(33)**	32.4 (13.6)

^a^Do you have... heart disease, stroke, heart failure, cancer, chronic obstructive pulmonary disease?

### Acceptability and satisfaction with the VBA intervention

The majority of patients reported they were asked and received advice about quitting smoking from their GP. It should be noted that four patients indicated that while their GP advised them to quit they did not ask them about their smoking status. We hypothesize that some GPs were aware of their patients smoking status (e.g. previous discussions, pack of cigarette visible, tobacco smoke odour) and as such did not ask the patient verbally.

Most patients were positive about being asked by their GPs about their smoking status, as well as being offered advice about smoking cessation. The majority of patients indicated that they expected their doctor to ‘ask’ and ‘advise’ them to quit as part of their practice duties.

*“I think that all doctors ask this... They are obliged to do so. I will say yes, I am satisfied with the doctor’s support. He is … .a good physician.” (Patient #407)*

*“ … I didn’t feel uncomfortable (with him asking and advising), I consider it as part of the medical history.” (Patient #403)*

*“He is doing his job.” (Patient #301)*

*“I thank him very much for asking and advising me. It shows he cares for me.” (Patient #402)*

Not receiving advice on smoking cessation was perceived by several patients as indicating a lack of concern on the part of GP for the patient’s wellbeing and poor professional practice.

*“I would say that she is not doing her job correctly if she didn’t ask me about smoking.” (Patient #106)*

*“It’s good that he cares and advises you to quit. Indifference from his side is not a positive thing.” (Patient #306)*

*“Someone has to care about it. It would show lack of interest if the doctor didn’t ask.” (Patient #308)*

Two participants from one of the GP practices surveyed reported their dislike with their GP’s advice about quitting smoking, noting that they had communicated their lack of interest in quitting at a recent consultation with the GP.

*“I didn’t like it (GP asking about smoking) … because I don't want to quit.” (Patient #107)*

*“I think the doctor should ask the people he sees but since he knows that some people don’t quit for years, he doesn’t have to ask every time she sees them.” (Patient #101)*

One of these participants (patient #101) noted that a patient’s low intention to quit smoking was a factor that should be taken into account when the doctor advises patients about smoking cessation counseling.

### Motivation to make a quit attempt and/or use available quit smoking treatments

The majority of patients indicated that they did not intend to quit smoking following the VBA intervention. One in four (*n* = 12/50) patients scheduled a follow-up appointment at the GP clinic to discuss smoking cessation (Table 2). A smaller number were prescribed a smoking cessation medication (6.0%, *n* = 3/50) or were referred to a quit smoking service (4.0%, *n* = 2/50). It would be important to note that data collection occurred immediately following the GP consult and it is possible that pharmacotherapy would be prescribed in a follow-up session for patients who were scheduled for a follow-up appointment with their GP to discuss smoking, or those referred to a specialized hospital-based quit smoking service/clinic.

**Table 2:** Patient’s report of VBA delivery at consultation (*n* = 50)

VBA Component	n (%)
**Ask**	44 (88.0)
**Advise**	48 (96.0)
**Act**	27 (54.0)
**Discussed quit smoking medications**	30 (60.0)
**Prescribed quit smoking medication**	3 (6.0)
**Scheduled follow-up appointment**	12 (24.0)
**Referred to quit smoking service**	2 (4.0)

Patients identified their GP’s active involvement in supporting their quit attempt to be a source of motivation and felt it would help them to quit smoking.

*“Yes, because it would be difficult (to quit) on my own, with the help of the doctor it would be better.” (Patient #304)*

*“The doctor’s support uplifts you psychologically and will help with quitting smoking.” (Patient #310)*

*“I have been thinking about quitting a great deal but I know I am not able to do it on my own. While now, this was a chance.” (Patient #507)*

Several patients spoke about the high level of trust they had for their doctor’s expertise and indicated that they would be pleased to accept counseling on smoking cessation from their GP now or in the future, as they felt their GP had the necessary knowledge and skills to provide effective support.

*“Very much (would be likely to seek the doctor’s support in the future), because he knows more than we do.” (Patient #310)*

Some patients also communicated a preference for receiving smoking cessation support from their GP, rather than be referred to other cessation services, noting trust and safety as the basis for this preference.

*“Yes, I want to quit but using the doctor’s help (versus referral to quit smoking clinic). I feel safer.” (Patient 309)*

*“No, no… I will stay here with the doctor. I have more trust here.” (Patient #507)*

### Barriers and facilitators to acting on VBA

Our analysis of patient barriers and facilitators to acting on VBA identified several themes, which we summarize here along with any patterns across patient sub-groups.

#### Perceived risk and expected benefits

Most of the patients who expressed an interest in quitting cited current health issues, while the expected health benefits from quitting smoking were the strongest factors affecting their decision to accept doctor’s advice and therapy.

*“ … it is a health issue. And because I have had an infarction, it is rational that I shouldn’t smoke.” (Patient #401)*

*“ … I want to quit and I always say I will, but I never manage. But now that I have a cold and dry mouth, which get worse with smoking, I will have to … ” (Patient #402)*

*“ … of course I would be interested, since I am actually having a health problem due to smoking.” (Patient #404)*

Several patients however identified that, given they were not currently experiencing any direct effects of their smoking on their health, they did not see any particular reason to quit smoking at this time.

*“Only if smoking created problems would I quit or expect the doctor to ask me … ” (Patient #107)*

#### Positive and supportive communication style

The majority of patients underlined the importance of a positive communication style when delivering VBA. Specifically identifying a preference for advice that showed concern for their well being. Some patients spoke about their feelings toward paternalist, aggressive or “fear-based” approaches they have experienced in the past noting this style was not effective in motivation them to think about quitting.

*“I expect the GP to ask in every visit about my smoking, as long as she doesn’t act like my mom.” (Patient #104)*

*“Yes, the doctor was helpful in the way he talked. He showed that he cared for me.” (Patient #309)*“*It is important for the doctor not to be aggressive. I mean you have a problem and you smoke … and then the doctor tells you (using a nasty voice) quit it … quit it...This becomes annoying.” (Patient #503)*

*“The way that he (the physician) will speak to me is important. I mean not to tell me quit smoking otherwise you will be sick in a year or two, your lungs will be damaged. This will scare me more.” (Patient #408)*

#### Self-efficacy and perceived importance of ‘will power’

Most patient participants expressed significant doubt about their ability to manage smoking cessation successfully specifically referencing that they are long standing smokers and that their smoking was a ‘personal weakness’. Several patients noted a lack of personal willpower as the key reason they would not be able to quit or act on their GP’s VBA.

*“I think about it very much but I can’t do it, because I have smoked for many years. The point is to achieve it (to quit). To become stronger than the cigarette.” (Patient #303)*

*“In the first place yes (was doctor’s advice helpful), but then, if you have been smoking for 20 years, it’s over.” (Patient #308)*

*“..the doctor may tell me today to quit and I have no problem (with him doing so), but don’t tell me to quit smoking entirely. This is my weakness.” (Patient #401)*

*“To my view, all these efforts are very nice but if you (the smoker) don’t put it into your mind, you cannot quit.” (Patient #306).*

*“It’s all about you, and if you want to quit … You need to want it as well.” (Patient #104)*

Several patients mentioned previous unsuccessful attempts as the reasons behind their reservation to engage in the process of smoking cessation and used them as strong arguments for a lack of confidence in their ability to act on VBA.

*“I have done so many things. I have done acupuncture, I didn’t manage (to quit). This is why I sometimes don’t pay attention, when they tell me to quit smoking. Because I think this is not for me.” (Patient #402)*

*“I have already visited it (smoking cessation clinic) in the past. It didn’t help me.” (Patient #505)*

#### Apprehension and fears about available smoking cessation interventions

Several patients identified significant reservation in terms of the effectiveness of available smoking cessation treatments. Some patients specifically identified concern about medication side effects as a factor that affects their interest in receiving counseling on smoking cessation and using treatments.

*“She mentioned that (about arranging an appointment to quit smoking) but I don’t think it will work for me.” (Patient #102) “The medicines scare me. I have no problems with the gum or patches, but other medicines-I have heard about needles and pills - These scare me to be honest.” (Patient #408)*

Multiple patients communicated a general disinterest in using medication of any sort as being the reason for not wanting to use quit smoking medications.

*“I don’t want to take medicines.” (Patient #106)*

*“I don’t want the medicines in general. I am not in favor of medicines.” (Patient #407)*

*“No I don’t want to take medicines. I will try to quit by myself.” (Patient #504)*

*“I want to avoid medicines. I already take medicines for another health issue and I don’t want to overload my body.” (Patient #506)*

#### Personal and financial resources

Several participants identified work and family responsibilities, a lack of time and financial resources, and stress as barriers to acting on their GP’s VBA to quit smoking. In particular, these were barriers related to being referred to a specialist smoking cessation clinic.

*“I don’t have someone to help me. I knew that there were some services but they are also a bit far away from where I live.” (Patient #402)*

*“Yes, but I will not be able to devote lengthy time for visits to the doctor (smoking cessation clinic) because I have a family and so on.” (Patient #309)*

*“There is no way (he laughs). I will have to pay for the doctor as well (smoking cessation clinic). And it will be very difficult.” (Patient #503)*

*“No way … (he laughs), I couldn’t deal with something more (being referred to a clinic or quitting smoking).” (Patient #504)*

## Discussion

Overall the VBA intervention was acceptable to smokers identified in general practice settings in Greece. Participants were positive about receiving VBA from their GP and considered it to be both part of the GPs clinical responsibilities, as well as an indication of the GP’s concern for their health. Following the VBA intervention, approximately one in four patients were referred to specialized smoking cessation service or scheduled a follow-up appointment to discuss quitting smoking further with their GP. While most patients who received VBA were not interested in quitting in the near future, several indicated their GP was a credible source of information and expertise and would access their support, should they decide to quit smoking in the future. There were a few patients, who were not interested in quitting smoking, who perceived the VBA intervention as bothersome and ineffective.

### Interpretation of results and implications to practice

Several patients spoke about the importance of the communication style used when delivering VBA. Specifically, patients identified that a communication style that conveyed caring and concern was more effective than the use of judgment, fear or lectures. This is consistent with previous research which highlights the importance of the tone in which cessation messages are communicated in engaging smokers in treatment [27]. Ensuring health care professionals are trained in effective techniques for delivering brief advice to smokers with an emphasis on techniques for providing non-judgmental supportive advice is important for improving effectiveness of VBA.

While a significant group of patients communicated a firm lack of interest in quitting smoking, others noted that they were aware that they should quit but did not feel confident in their ability to do so despite the supports that are available. Some patients communicated their belief that there was no reason to quit until they had a serious health issue. This may suggest that patients who have yet to experience a smoking-related illness may not accurately understand the risk associated with smoking and importance of quitting. Not surprisingly, previous research has shown that GPs also tend to advise patients with smoking-related illness more frequently and with greater persistence than healthy patients [28]. It is not known if the increased frequency of GP advice to patients with a smoking-related illness is independent of patients’ beliefs about risk or if there is an association. Given the importance of smoking cessation in preventing major chronic diseases, exploring simple strategies for enhancing the impact of VBA interventions among individuals who have not yet developed a smoking-related illness may increase the reach of this intervention among healthy smokers who are often younger in age. This is particularly important, given that the majority (75%) of smokers who participated in the study did not have a smoking related illness but did report heavy smoking over an extended time period.

Several patients who were not ready to quit identified a lack of ‘willpower’ as being the main reason for not being able to act on the VBA intervention received from their GP. Specifically participants communicated the belief that quitting was a ‘matter of the mind’ and that quitting is ‘best done on one’s own’. There was also reluctance among a large segment of participants to use of available quit smoking medications and very low interest in being referred to the available hospital-based quit smoking clinic. Patients referenced willpower in association with comments regarding the belief that the medication and counseling support was unlikely to be effective for them. This may suggest a lack of understanding among patients about nicotine addiction and such gaps in understanding may reduce the likelihood that patient will use pharmacotherapy or counseling. These findings are consistent with research by Hughes et al., which reported on the role of patient’s perspective in terms of their beliefs of the role of willpower, self-efficacy and use of treatment in supporting smoking cessation [14, 29]. The study found that a large proportion (60–78%) of smokers believe that willpower is ‘necessary’ but also ‘sufficient’ for quitting and that this belief appears to undermine quit attempts as fewer quit attempts were reported among smokers with this belief. Other studies have reported similar findings [30–32]. Examining techniques for reframing the role of willpower to smokers and providing information on nicotine addiction may be an important target for future research in order to enhance the effectiveness of VBA. It is important when interpreting results to acknowledge that in contrast to countries such as the UK, Australia, Canada where there has been great success in term of the social denormalization of smoking, Greece has lagged behind other EU countries. At the time of this study many workplaces and public spaces in Greece did not enforce smoke-free bans and as such it is very common to see on a regular basis smoking in these environments. The socio-cultural norms related to smoking in Greece are expected to directly affect a smoker’s self-efficacy with quitting, and impede patient from acting on VBA and using evidence-based cessation supports [33, 34]. In fact, European monitoring surveys have repeatedly documented low intentions to quit among smokers in Greece compared to other EU countries [35, 36]. Additionally, participants in the present study were heavy smokers, with high rates of nicotine addiction and had been smoking for a very long-time, which may contribute to low confidence in quitting. Importantly, the smoking profile of patients sampled is similar to that reported in national surveys for the general population of smokers in Greece [37].

Many patient participants in the present study expressed concern about available quit smoking services with some patients identifying them as being costly, located at a distance (i.e. in hospital within only the larger city centres), chaotic, or not appropriate for their personal situation. Several patients had personal experience with the use of hospital-based smoking cessation clinics to speak from and others simply had the perception of these barriers. Additionally, most patients indicated a preference for quitting with the support of their GP.

Our results are similar to findings from a recent examination of barriers to accessing support in England [16]. Specifically this study found work and time constraints, the belief that smokers should quit on their own and that nothing can help with quitting smoking, and low motivation to quit were among the top barriers to not accessing available stop smoking supports. The study found that reported barriers differed based on patient readiness to quit, self-efficacy with quitting and age. Other studies have found greater heaviness of smoking predicted the use of assistance [38] Findings from our study and that of others are consistent with the COM-B model of behaviour change which highlights the role of motivation, capabilities (self-efficacy), opportunities as central to changing smoking behaviour.

### Impact of the study

Given the high rates of tobacco use, low intentions to quit, and lack of policy supports there is reason to believe the VBA intervention may not be as impactful among smokers in Greece compared to the UK. Despite this, one in four patients were documented to have accepted the opportunity to receive further support with quitting from their GP or be referred to a hospital-based quit smoking clinic, which would be considered a positive outcome for a low-cost intervention such as VBA. In order to further increase the likelihood of patients making a quit attempt, VBA may be coupled with other office-based smoking cessation interventions such as ‘reduce to quit’ approaches to cessation and motivational interventions such as motivational interviewing which have been shown to increase likelihood of quitting among patients not ready to quit [26, 39, 40].

The VBA intervention is intended to work synergistically with specialized smoking cessation services to whom GPs and other HCPs can refer patients ready to quit to receive evidence-based counseling and pharmacotherapy to support cessation. In the UK there is a well-developed network of local quit smoking services who deliver cessation treatment to patients within their communities and often have strong ties to general practice. In countries such as Greece, where there is a less developed smoking cessation system, barriers to access may impede patients from acting on VBA and accessing evidence-based cessation supports. When planning VBA interventions and service delivery models issues such as access and patient preference to services delivered by trusted GPs should be considered.

### Study limitations

The results of this study should also interpreted in light of its limitations. The qualitative nature of the present study has significant strengths in terms of understanding the patient perspective, however a quantitative evaluation may also be useful in generating data on the frequency of the reported barriers and facilitators to acting on VBA in a broader sample of tobacco users. This study was limited to data from a sample of 50 patients who smoked recruited from five GP practices and are as such a reflection of these individual’s perceptions and experience. Future research may wish to validate findings in a broader sample of practices and patients. Given non-participants reported lower daily cigarette consumption our results may be less representative of smokers with lower levels of dependence. Sixty per cent of participants resided in rural areas with no local access to specialized cessation services and comments should be interpreted with that understanding. This study was conducted in Greece and it is not clear the extent to which study findings would be generalizable to other countries, although it is likely there would be application to other Southern European countries as well as countries with limited cessation supports.

## Conclusions

The majority of respondents reported high levels of acceptability and satisfaction with the VBA intervention. Patients confirmed the motivational role of advice from GPs. GPs should expect that a small minority of patients in particular those who are not interested in quitting and with whom they may have previously discussed quitting smoking will not be receptive. The important role of a supportive and caring tone in the delivery VBA was highlighted. Patient views on the role of personal health risks of smoking, ‘will power’, and apprehension about available quit smoking supports appear to affect patient’s motivation to act on VBA and should be targets for the design and implementation of future VBA interventions. Data generated from this study offers insights, which may be particularly applicable to countries such as Greece who have high rates of tobacco use and limited cessation supports.

## Data Availability

The datasets used and/or analysed during the current study are available from the corresponding author upon request.

## Abbreviations

VBA: Very Brief Advice on Smoking
GP: General practitioner
NCSCT: National Centre for Smoking Cessation and Training

## References

[1] World Health Organization. WHO report on the global tobacco epidemic 2019: offer help to quit tobacco use. Geneva: World Health Organization; 2019. ISBN 978-92-4-151620-4. Available from: https://www.who.int/tobacco/global_report/en/.

[2] World Health Organization European Region Office. Roadmap of actions to strengthen implementation of the WHO framework convention on tobacco control in the European region 2015–2025: making tobacco a thing of the past. Geneva; World Health Organization: 2015. Available from: https://www.euro.who.int/__data/assets/pdf_file/0005/297563/WHO-Roadmap-report-tobacco-control-15-25-en.pdf.

[3] Hummel K, Nagelhout GE, Fong GT, Vardavas CI, Papadakis S, Herbec A (2018). Quitting activity and use of cessation assistance reported by smokers in eight European countries: findings from the EUREST-PLUS ITC Europe surveys. Tob Induc Dis.

[4] European Commission. Special Eurobarometer 458: Attitudes of Europeans towards tobacco and electronic cigarettes. Brussels: European Commission;2017. ISBN 978-92-79-69104-1.

[5] Stead LF, Buitrago D, Preciado N, Sanchez G, Hartmann-Boyce J, Lancaster T (2013). Physician advice for smoking cessation. Cochrane Database Syst Rev.

[6] Aveyard P, Begh R, Parsons A, West R (2012). Brief opportunistic smoking cessation interventions: a systematic review and meta-analysis to compare advice to quit and offer of assistance. Addiction.

[7] Forum NF (2012). The NHS’s role in the public’s health: a report from the NHS future forum.

[8] Royal College of Physicians (RCP) (2018). Hiding in plain sight: treating tobacco dependence in the NHS.

[9] Stead LF, Koilpillai P, Fanshawe TR, Lancaster T (2016). Combined pharmacotherapy and behavioural interventions for smoking cessation. Cochrane Database Syst Rev.

[10] Vogt F, Hall S, Marteau TM (2010). Examining why smokers do not want behavioral support with stopping smoking. Patient Educ Couns.

[11] Morphett K, Partridge B, Gartner C, Carter A, Hall W (2015). Why don’t smokers want help to quit? A qualitative study of smokers’ attitudes towards assisted vs. unassisted quitting. Int J Environ Res Public Health.

[12] Vogt F, Hall S, Marteau TM (2008). Understanding why smokers do not want to use nicotine dependence medications to stop smoking: qualitative and quantitative studies. Nicotine Tob Res.

[13] Hughes JR, Marcy TW, Naud S (2009). Interest in treatments to stop smoking. J Subst Abus Treat.

[14] Hughes JR (2009). Smokers’ beliefs about the inability to stop smoking. Addict Behav.

[15] Roddy E, Antoniak M, Britton J, Molyneux A, Lewis S (2006). Barriers and motivators to gaining access to smoking cessation services amongst deprived smokers--a qualitative study. BMC Health Serv Res.

[16] Kale D, Gilbert H, Sutton S (2019). An exploration of the barriers to attendance at the English stop smoking services. Addict Behav Rep.

[17] Twyman L, Bonevski B, Paul C, Bryant J (2014). Perceived barriers to smoking cessation in selected vulnerable groups: a systematic review of the qualitative and quantitative literature. BMJ Open.

[18] Michie S, Churchill S, West R (2011). Identifying evidence-based competences required to deliver behavioural support for smoking cessation. Ann Behav Med.

[19] Michie S, van Stralen MM, West R (2011). The behaviour change wheel: a new method for characterising and designing behaviour change interventions. Implement Sci.

[20] Joossens LRM. The tobacco control scale. Monitoring the implementation of tobacco control policies systematically at country-level across Europe: European Cancer Leagues Association & Catalan Institute of Oncology; 2016. Available at: https://www.tobaccocontrolscale.org/.

[21] Girvalaki C, Papadakis S, Vardavas C, Petridou E, Pipe A, Lionis C (2018). Smoking cessation delivery by general practitioners in Crete, Greece. Eur J Public Health.

[22] Tong A, Sainsbury P, Craig J (2015). Consolidated criteria for reporting qualitative research (COREQ): a 32-item checklist for interviews and focus groups. Int J Qual Health Care.

[23] Barker F, Atkins L, de Lusignan S (2016). Applying the COM-B behaviour model and behaviour change wheel to develop an intervention to improve hearing-aid use in adult auditory rehabilitation. Int J Audiol.

[24] McEwen AP J, Lionis C, Papadakis S, Tsiligianni I, Anastasaki M, An PL, Vinh NN, Binh P, Quỳnh N, Le H, Talant S, Ayzhamal DT, Beyshenbekova A, Marajopov N, Sheraliev U (2019). Adapting very brief advice (VBA) on smoking for use in low resource settings: experience from the FRESH AIR project. J Smok Cessat.

[25] Patton M (1990). Qualitative evaluation and research methods.

[26] Klemperer EM, Hughes JR, Callas PW, Solomon LJ (2017). A mediation analysis of motivational, reduction, and usual care interventions for smokers who are not ready to quit. Nicotine Tob Res.

[27] Marcano Belisario JS, Bruggeling MN, Gunn LH, Brusamento S, Car J (2012). Interventions for recruiting smokers into cessation programmes. Cochrane Database Syst Rev.

[28] Papadakis S, Girvalaki C, Vardavas C, Pipe A, Cole A, Tsiligianni I (2018). Factors associated with rates of tobacco treatment delivery by General Practitioners in Greece: Missed opportunities for prevention?. Toba Induc Dis.

[29] Hughes JR, Naud S (2016). Perceived role of motivation and self-efficacy in smoking cessation: a secondary data analysis. Addict Behav.

[30] Cook-Shimanek M, Burns EK, Levinson AH (2013). Medicinal nicotine nonuse: smokers’ rationales for past behavior and intentions to try medicinal nicotine in a future quit attempt. Nicotine Tob Res.

[31] Balmford J, Borland R (2008). What does it mean to want to quit?. Drug Alcohol Rev.

[32] Tzelepis F, Paul CL, Walsh RA, Knight J, Wiggers J (2013). Who enrolled in a randomized controlled trial of quitline support? Comparison of participants versus nonparticipants. Nicotine Tob Res.

[33] Konstantopoulou SS, Behrakis PK, Lazaris AC, Nicolopoulou-Stamati P (2014). Indoor air quality in a bar/restaurant before and after the smoking ban in Athens, Greece. Sci Total Environ.

[34] Filippidis FT, Tzoulaki I (2016). Greece giving up on tobacco control. Addiction.

[35] European Commission. Special Eurobarometer 458. Attitudes of Europeans towards tobacco and electronic cigarettes. Brussels: European Commission; 2015. ISBN 978-92-79-48050-8.

[36] Schoretsaniti S, Filippidis FT, Vardavas CI, Dimitrakaki C, Behrakis P, Connolly GN (2014). 5-year trends in the intention to quit smoking amidst the economic crisis and after recently implemented tobacco control measures in Greece. Addict Behav.

[37] Filippidis FT, Vardavas CI, Loukopoulou A, Behrakis P, Connolly GN, Tountas Y (2013). Prevalence and determinants of tobacco use among adults in Greece: 4 year trends. Eur J Pub Health.

[38] Myers MG, Strong DR, Linke SE, Hofstetter CR, Al-Delaimy WK (2015). Predicting use of assistance when quitting: a longitudinal study of the role of quitting beliefs. Drug Alcohol Depend.

[39] Lindson-Hawley N, Hartmann-Boyce J, Fanshawe TR, Begh R, Farley A, Lancaster T (2016). Interventions to reduce harm from continued tobacco use. Cochrane Database Syst Rev.

[40] Lindson-Hawley N, Thompson TP, Begh R (2015). Motivational interviewing for smoking cessation. Cochrane Database Syst Rev.

